# Soluble CD14 as a Diagnostic and Prognostic Biomarker in Hematological Patients with Febrile Neutropenia

**DOI:** 10.1155/2017/9805609

**Published:** 2017-08-06

**Authors:** Sini Korpelainen, Carina Intke, Sari Hämäläinen, Esa Jantunen, Auni Juutilainen, Kari Pulkki

**Affiliations:** ^1^University of Eastern Finland and Eastern Finland Laboratory Centre, Kuopio, Finland; ^2^Kuopio University Hospital, Kuopio, Finland; ^3^University of Eastern Finland and Kuopio University Hospital, Kuopio, Finland

## Abstract

**Objective:**

Elevated levels of a cell surface glycoprotein, soluble cluster of differentiation 14 (sCD14), have been observed in patients with sepsis. Only scarce data are available on sCD14 in hematological patients with chemotherapy-induced febrile neutropenia. The study aim was to investigate sCD14 as an early biomarker in febrile neutropenia after intensive chemotherapy to detect a rapidly deteriorating clinical course early enough to avoid serious infectious complications.

**Patients and Methods:**

This prospective study included 87 adult hematological patients at the start of febrile neutropenia after intensive chemotherapy for acute myeloid leukemia or after autologous stem cell transplantation. The study endpoints were septic shock, severe sepsis, and positive blood culture findings. sCD14 was analyzed from day 0 to day 2, and its prognostic capacity was compared to that of C-reactive protein and procalcitonin.

**Results:**

Plasma level of sCD14 predicted the development of septic shock on day 1 (*p* = 0.001) and day 2 but not the development of severe sepsis or blood culture positivity in hematological patients with chemotherapy-induced febrile neutropenia.

**Conclusions:**

Soluble CD14 did not predict an overall complicated course at the early stages of febrile neutropenia. However, it was helpful in predicting the progression of the clinical course of neutropenic fever to septic shock.

## 1. Introduction

Patients receiving intensive chemotherapy for hematological malignancies frequently develop febrile neutropenia. They are at high risk of sepsis and septic complications. The onset of septic infection can be insidious, and the outcome may be fatal. An early diagnosis of sepsis is crucial in the prevention of serious complications, and it still remains a challenge for clinicians because of the lack of appropriate diagnostic methods [1]. By definition, demonstrating bacteremia requires blood cultures, but they are time-consuming and often give false-negative results or get microbial contamination [2]. C-reactive protein (CRP) is widely used as an indicator of infection, but it reacts too slowly for prognostic use at the early stages of sepsis [3]. Procalcitonin (PCT) has been studied in neutropenic patients and found to be useful in distinguishing sepsis from noninfectious causes of fever [4, 5]. One of the new biomarkers for sepsis is soluble CD14 [6], but there are limited data on its predictive value in febrile neutropenia of adult patients with hematological malignancies.

Cluster of differentiation 14 (CD14) is a cell surface glycoprotein expressed mainly in innate immune response cells such as monocytes, macrophages, neutrophils, and B cells. CD14 recognizes ligands at the cell surface of both gram-negative and gram-positive bacteria and binds to them. It serves as a high-affinity receptor for lipopolysaccharide (LPS) and LPS-binding protein (LBS) complexes. The CD14-LPS-LBS-complex activates the Toll-like receptor 4-specific proinflammatory signalling cascade against infectious agents. The complex including CD14 is released from the cell membrane into the circulation creating soluble CD14 (sCD14) [7–9]. Soluble CD14 can also be directly released by hepatocytes [10]. Circulating plasma proteases modify soluble CD14 to yet another molecule, soluble CD14 subtype (sCD14-st) or presepsin [7].

Soluble CD14-st (presepsin) binds to bacterial products while circulating in the peripheral blood. It introduces these products to epithelial and endothelial cells, contributing to the activation of the cells against bacteria. Elevated levels of sCD14 have been found in patients with sepsis in several clinical conditions. In previous studies, levels of sCD14 have been associated with sepsis mortality [11–19]. In particular, Burgmann et al. reported that increased levels of serum sCD14 were associated with a high mortality in gram-positive sepsis [12].

Also, soluble CD14-st (presepsin) has been considered as a promising biomarker because of its rapid increase at the early stages of sepsis and the availability for bed-side analysis. However, also other than bacteria-related conditions influence the level of sCD14-st, which poses a challenge for its diagnostic use. When used as the only diagnostic method, sCD14-st did not show any advantage over PCT in distinguishing sepsis from nonsepsis [6, 8, 20, 21].

There are only few data on sCD14 in patients with chemotherapy-induced febrile neutropenia at risk for complicated course of sepsis. Urbonas et al. [22] evaluated the predictive value of soluble CD14-st and reported that no association was observed between sCD14-st and bacteremia or sepsis in chemotherapy-induced febrile neutropenia in pediatric patients. According to Urbonas et al., reduced amount of innate immune cells due to chemotherapy may have influenced the results of the study [22]. Also, Olad et al. observed that among neutropenic pediatric cancer patients, sCD14 was not useful in the detection of bacteremia, although sCD14 levels were elevated in blood culture-positive patients, when a clinically detectable source of infection was absent [23].

Data concerning sCD14-st and adult hematological patients at risk for complicated course for febrile neutropenia are especially scarce and controversial. In a study including adult hematological patients with febrile neutropenia, sCD14-st levels were higher in patients with septic shock than in those without it. The study consisted of two cohorts. In the first cohort, the results did not quite gain statistical significance in spite of the positive tendency. Based on the results of the latter cohort, sCD14-st was an earlier and more sensitive indicator of bacterial infection than PCT, but the number of cases was very small [24].

The aim of this study was to evaluate sCD14 as a predictor of the progression of febrile neutropenia to sepsis and its complications and to compare sCD14 with other sepsis biomarkers such as CRP and PCT in hematological patients.

## 2. Patients and Methods

The study population consisted of adult patients treated on the hematology ward of Kuopio University Hospital between November 2009 and November 2012. All patients who were treated for acute myeloid leukemia (AML) or who were autologous stem cell transplant (ASCT) recipients receiving intensive chemotherapy were invited to participate in the study. The final inclusion criteria were fulfilled if a patient had febrile neutropenia. The study population consisted of 87 patients, 23 with AML, and 64 of them were ASCT recipients. The median age was 61 years (range 18–70 years). Further patient characteristics are shown in Table 1.

All study patients were carefully followed up until recovery of neutropenia (median 7 days, range 4–35 days). Blood pressure, oxygen saturation, respiratory frequency, heart rate, skin temperature, urine output, and fluid intake were followed up. Each patient was daily examined thoroughly for clinical signs and sources of infections. Attention was paid to features indicating the development of severe sepsis or septic shock. Broad spectrum antibiotics were started for all patients after the samples for blood cultures were taken at the onset of fever. Antibacterial treatment was adjusted according to blood culture results, radiological and clinical findings. For patients receiving ASCT for lymphoma, ciprofloxacin-prophylaxis was used. Altogether, 56 out of 87 (64%) patients received granulocyte colony-stimulating factor in order to shorten the length of neutropenia.

### 2.1. Study Definitions

A single positive blood culture was considered significant if the microbe was a clinically relevant cause of infection. Common skin contaminants (e.g., coagulase-negative staphylococci) were considered significant only if they were found in two consecutive blood cultures or if there was a concurrent skin or catheter infection.

*Febrile neutropenia* was defined using the criteria of the Infectious Diseases Society of America [25]. Neutropenia was defined as a neutrophil count less than 0.5 × 10^9^/L or a count less than 1 × 10^9^/L with a predicted decrease to less than 0.5 × 10^9^/L. Fever was defined as a single oral temperature of 38.3°C or over or a temperature of 38.0°C or over for 1 h or more.

*Sepsis and septic shock were defined* according to the guidelines of the American College of Chest Physicians Consensus [26, 27]. *Sepsis* was defined as a syndrome in which systemic inflammatory response was present with infection diagnosed clinically or microbiologically. *Septic shock* was defined as a subset of sepsis, if hypoperfusion (systolic arterial pressure < 90 mmHg, a mean arterial pressure < 60 mmHg, or a reduction in systolic blood pressure of >40 mmHg from baseline) was present despite adequate volume resuscitation in the absence of other causes of hypotension. *Severe sepsis* was defined as a subset of sepsis with sepsis-induced organ dysfunction [28]. *Complicated course of febrile neutropenia* was defined as a positive blood culture finding and/or development of severe sepsis or septic shock during the period from the onset of febrile neutropenia until the recovery of neutropenia. In February 2016, new definitions for sepsis and septic shock were announced including the Sequential Organ Failure Assessment (SOFA) and Quick SOFA [29], but in this prospective study design, we used the definitions that were available at the time of the study entry. However, for comparison, also Quick SOFA was retrospectively calculated according to the new sepsis definition [29].

### 2.2. Blood Cultures

Blood cultures were processed using the automated blood culture system Bactec 9240 (Becton Dickinson, Sparks, MD, USA). The incubation episode was 7 days for both aerobic and anaerobic bottle and 42 days for MYCO F/Lytic bottles.

### 2.3. Sample Collection and Laboratory Analysis

Blood samples for sCD14, CRP, and PCT analyses were collected at the onset of febrile neutropenia (day 0) and the further samples in the following two mornings (day 1 and day 2). Serum samples were stored frozen at −70°C until analyzed.

The concentration of sCD14 was measured with an ELISA kit (R&D Systems, Minneapolis, MN, USA). The minimum detectable concentration of sCD14 in this assay was 125 ng/L. The respective intra- and interassay CVs for sCD14 analyses were 10.7% and 16.0% for 1285 ng/L and 1549 ng/L of sCD14 and 6.1% and 6.4% for 1430 ng/L and 1561 ng/L of sCD14.

The concentration of CRP was measured with a Konelab 60i clinical chemistry analyzer (Lab systems CLD, Konelab, Helsinki, Finland) or Cobas 6000 analyzer (Hitachi, Tokyo, Japan). The between-run variations were from 2.3% to 4.3%. The upper reference limit of serum or plasma CRP of a healthy reference population is 5 mg/L.

Plasma PCT was analyzed with a Cobas 6000 analyzer (Hitachi, Tokyo, Japan). The sensitivity of the assay was 0.06 *μ*g/L. The respective within- and between-assay CVs for PCT analyses were 1.4% and 3.0% for 0.46 *μ*g/L of PCT and 1.1% and 2.6% for 9.4 *μ*g/L of PCT. The reference limit for PCT indicating a possible systemic infection is 0.5 *μ*g/L.

### 2.4. Statistical Analysis

Data analyses were conducted with SPSS version 23 for Windows (SPSS, Chicago, IL, USA). Categorical variables like groups defined by the endpoints were given as absolute counts and percentages. Correlations between variables were analyzed with Pearson's correlation test when appropriate or with nonparametric Spearman's correlation test. The Mann–Whitney *U* test or Kruskal-Wallis test was used to evaluate the differences in sCD14 concentration levels between patient groups. The difference between sCD14 on day 0 and on day 1 and sCD14 on day 1 and on day 2 was analyzed with related-samples Wilcoxon signed-rank test. Receiver operating characteristic (ROC) curve analysis was performed to compare and describe the diagnostic ability between sCD14, PCT and CRP. A *p* value of less than 0.05 was considered significant.

### 2.5. Ethics

The study was conducted in accordance with the current version of the Helsinki Declaration. Informed consent was obtained from all individual participants included in the study. The study has the permission of the Ethical Board of Kuopio University Hospital (100/2006).

## 3. Results

Positive blood cultures were observed in 18 patients with febrile neutropenia (21%), and three of these patients (17%) developed septic shock (two with *Enterococcus faecium* and one with *Pseudomonas aeruginosa*). The blood cultures showed gram-negative bacteria in three patients (17% out of positive blood culture findings), gram-positive bacteria in 14 patients (78%), and a fungus in one patient (5%). The gram-negative findings included *Escherichia coli* (*n* = 1), *Klebsiella oxytoca* (*n* = 1), and *Pseudomonas aeruginosa* (*n* = 1). The gram-positive findings included *Enterococcus faecium* (*n* = 5), *Staphylococcus epidermidis* (*n* = 3), *Staphylococcus haemolyticus* (*n* = 2), *Streptococcus mitis* (*n* = 2), *Gemella morbillorum* (*n* = 1), and *Streptococcus salivarius* (*n* = 1). The only fungal finding was *Candida krusei*. Altogether, eight patients (9.2%) developed severe sepsis, and twenty patients (23.0%) fulfilled the criteria for complicated course of febrile neutropenia. Three patients developed septic shock, and two of these three patients died during the hospital stay. Altogether, three patients died during the hospital stay.

When analyzed retrospectively, 7/8 patients with severe sepsis had 1 point in quick SOFA scoring not predicting high risk for in-hospital mortality. Six of these patients were treated at the intensive care unit (ICU), and two of them developed septic shock leading to the death of one of them. Only 1/8 patients received 3 quick SOFA points predicting high risk for in-hospital mortality. This patient developed septic shock and died at the ICU.

The distributions of some of the continuous variables were not normal, so nonparametric tests were used. Data were expressed as medians with interquartile ranges or ranges from minimum to maximum. In all patients, the median serum sCD14 concentration (interquartile range) was 1428 (1088–1888 ng/L), 1548 (1084–1992 ng/L), and 1626 (1179–2005 ng/L) on days 0, 1, and 2, respectively. Median CRP level (interquartile range) was 38 (19–69 mg/L), 73 (41–119 mg/L), and 103 (45–178 mg/L) on days 0, 1, and 2, respectively, and the median serum PCT concentration (interquartile range) was 0.138 (0.090–0.212 *μ*g/L), 0.185 (0.109–0.408 *μ*g/L), and 0.208 (0.104–0.547 *μ*g/L) on days 0, 1, and 2, respectively. There was no statistically significant increase in the level of sCD14 concentration from day 0 to day 1 or from day 1 to day 2.

sCD14 concentration did not correlate with CRP or PCT concentration on day 0, day 1, or day 2. The duration of neutropenia did not correlate with sCD14 on any day. Age, sex, comorbidities, or the type of hematological malignancy had no statistically significant association with the levels of sCD14 on days 0, 1, or 2. sCD14 level had a slight positive correlation with the amount of leukocytes on day 2 (Pearson's correlation coefficient 0.233 with *p* value 0.036 for leukocytes).

**Figure 1:**
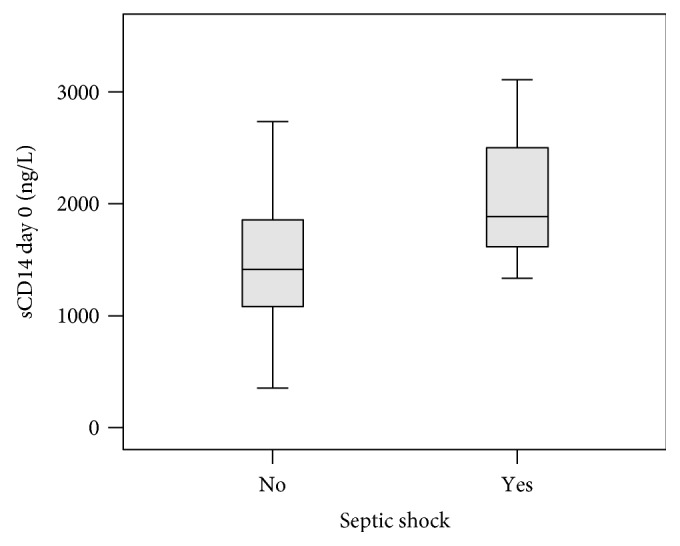
Boxplot graph showing sCD14 concentration levels on day 0 in patients with and without developing septic shock (*p* value 0.176). The horizontal bold line represents median, the box interquartile range, and the end of the lines minimum and maximum values.

**Figure 2:**
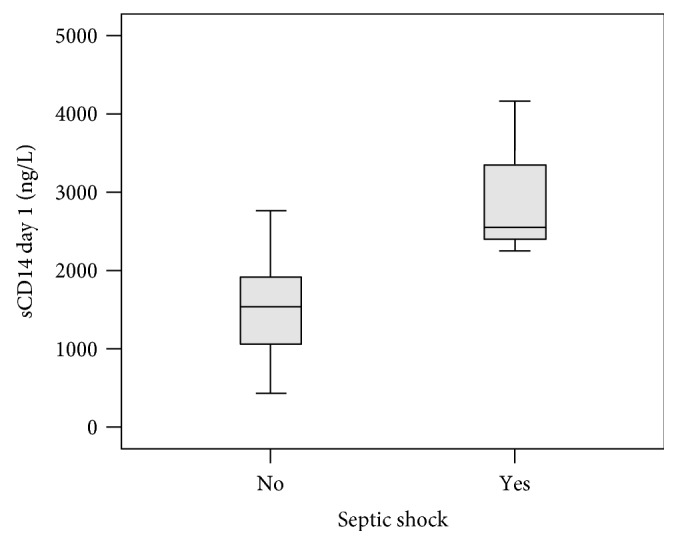
Boxplot graph showing sCD14 concentration levels on day 1 in patients with and without developing septic shock (*p* value 0.001). The horizontal bold line represents median, the box interquartile range, and the end of the lines minimum and maximum values.

The mean and median of sCD14 concentration on days 1 and 2 in those patients who developed septic shock were higher in comparison to those in patients who did not develop septic shock (Table 2, Figures 1 and 2). The values on day 1 were the highest. The patient with the highest levels of sCD14 (3110 ng/L, 4150 ng/L, and 3730 ng/L on days 0, 1, and 2, resp.) developed septic shock, dying during the hospital stay on the 12th day after the onset of the fever. The second patient who developed septic shock had levels of sCD14 1890 ng/L, 2250 ng/L, and 2060 ng/L on days 0, 1, and 2, respectively. He recovered from sepsis. The third patient had a rising value of sCD14 on days 0 and 1 (1340 ng/L–2550 ng/L). The patient died before day 2; blood samples were not collected on that day.

Although the levels of sCD14 were higher in patients with clinical course progressing to septic shock than in those not with clinical course progressing to septic shock, the patients with positive and negative blood culture findings had similar levels of sCD14 (Table 3), whether the microbe was gram-positive or gram-negative (data not shown). In comparison, plasma levels of CRP on day 1 and PCT on day 0, day 1, and day 2 were higher in blood culture-positive than in blood culture-negative patients.

Furthermore, sCD14 levels at the beginning of febrile neutropenia were not associated with the development of severe sepsis (Table 4). In comparison, elevated levels of CRP on day 1 and day 2 and of PCT from day 0 to day 2 were associated with the development of severe sepsis.

**Figure 3:**
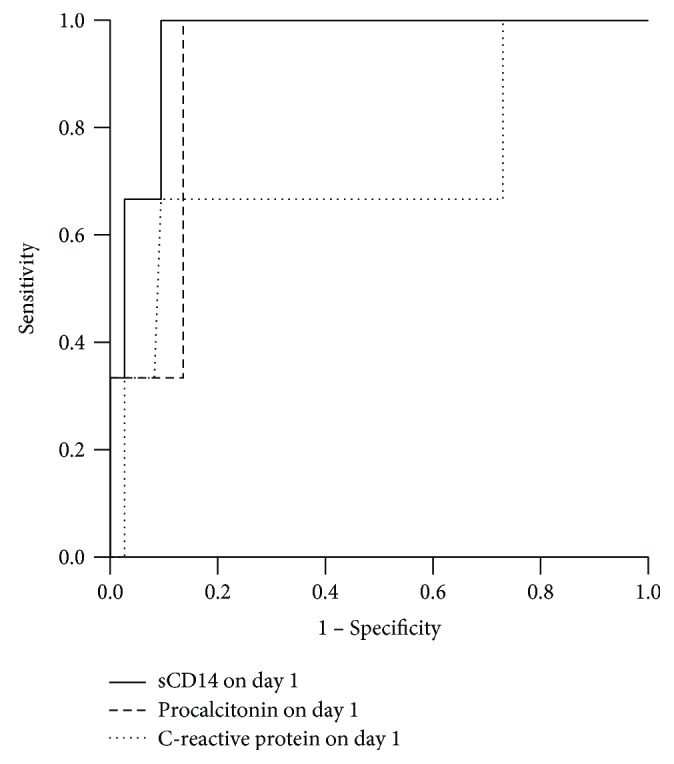
Receiver operating characteristic (ROC) curve analysis comparing sCD14 (continuous line), procalcitonin (sparsely dotted line) and C-reactive protein (CRP) (densely dotted line) predicting the development of septic shock on d1. For sCD14, the area under the curve (AUC) was 0.959 (95% CI 0.902–1.000) with SD 0.029 and *p* value 0.007 to differentiate patients developing septic shock. For procalcitonin, the AUC was 0.910 (0.819–1.000) with SD 0.047 and *p* value 0.017. For CRP, the AUC was 0.716 (0.354–1.000) with SD 0.186 and *p* value 0.202 (nonsignificant), respectively.

However, sCD14 predicted septic shock on day 1 (with area under the curve (AUC) 0.959, 95% CI 0.902–1.000, and *p* value 0.007) showing a higher AUC for sCD14 than that for CRP or PCT in regard to the development of septic shock (Figure 3).

## 4. Discussion

In this three-year prospective study, soluble CD14 did not show any significant association with blood culture positivity or with severe sepsis, but high levels of soluble CD14 on the first and second days of neutropenic fever were associated with the development of septic shock in neutropenic hematological patients who had received intensive chemotherapy.

The strengths of this study were a homogenous study population of neutropenic hematological adult patients, precise timing of sampling, and the prospective data collection. The main limitation of this study was the small number of patients with septic shock or death. The focus in our study was in pre-emptive patient care, including the aim of early recognition of sepsis. Therefore, the old definition for sepsis, also as the only definition enforced during the study enrolment, was applied [26, 27]. Retrospectively, we also evaluated Quick SOFA for sepsis definition, but limitations were recognized, as also described by Sprung et al. [30], who critically reviewed the new definition for sepsis based on the problems met in early sepsis recognition and in retrospective derivation for the SOFA score [30]. According to Sprung et al., “the new definitions discard the sepsis spectrum.” Our findings indicate that neutropenic sepsis may be one of the sepsis types that are discarded.

Several new biomarkers have been studied for their ability to predict the development of febrile neutropenia to sepsis, but so far, none of them has shown to be superior to traditional diagnostic methods and, therefore, has not been implemented in routine clinical use. Previously, we have discussed the possible impact of the lack of neutrophils on biomarker levels in hematological patients after intensive chemotherapy. The precise influence of neutropenia on the kinetics of a biomarker is usually indeterminate [3, 31–38]. In the present study, a slight but statistically significant positive correlation was observed between the leukocyte count and the levels of sCD14, possibly partly accounting for the overall weak associations between sCD14 levels with the outcomes, as in the presence of low leukocyte count also low levels of sCD14 levels are encountered. Chemotherapeutic agents also influence the function and number of other leukocytes and, therefore, other sources of sCD14 production. This may have further weakened the association of sCD14 levels with blood culture positivity in hematological patients after intensive chemotherapy. In addition to leukocytes, hepatocytes also produce sCD14 [10, 14]. There are also genetic variations in CD14 expression, which influence mCD14 as well as sCD14 levels in serum, but the impact of CD14 expression on the clinical picture of septic infection is unclear [18].

As soluble CD14 is an acute phase protein introducing bacterial products to leukocytes and other cells at the early stage of an infection, it may prevent lethal side effects of bacterial products at the late phase of an infection [17, 39]. High concentrations of soluble CD14 as well as its subtype are also associated with impaired kidney function, which may explain the elevated values in patients with septic shock [40–44]. In combination with other diagnostic methods, sCD14 concentration may be helpful to find neutropenic hematological patients at high risk for potentially lethal septic shock, but further studies in larger patient groups are needed to confirm these preliminary findings.

Sepsis as such is a complicated and multifactorial process with a plethora of potential biomarkers. Typically, sepsis biomarkers are affected by the type of pathogen, comorbidities, genetic variation, medication, and host response. The search for a new biomarker to detect sepsis remains active as there is a constant need to improve the current diagnostic methods in clinical use. The recognition of septic infections is crucial at the onset of neutropenic fever to guide the therapy and to improve the outcome. There is a need to study additional factors, such as the role of underlying diseases, chemotherapeutic regimens, and the influence of neutropenia on blood levels and kinetics of a specific biomarker in patients with chemotherapy-induced neutropenia.

In conclusion, soluble CD14, measured in the beginning of febrile neutropenia after intensive chemotherapy in hematological patients, appeared to be a helpful biomarker in predicting progression of the clinical course to septic shock. However, it did not predict blood culture positivity or severe sepsis and was inferior to PCT and CRP in predicting these latter two outcomes.

## Figures and Tables

**Table 1:** Characteristics of the study population (*n* = 87).

General	
Male (*n*, %)	55 (63%)
Age (years, median, range)	61 (18–70)
Age over 60 years (*n*, %)	47 (54%)
Diagnosis (*n*, %)	
AML	23 (26%)
ASCT recipients	64 (74%)
NHL	43
MM	18
HL	3
Chemotherapy regimen (*n*)	
BEAM	42
HD-MEL	18
IdAraC-Ida	9
IAT	7
MEA	4
Carmustine-thiotepa	3
Mito-HDAraC	2
HDAraC-Ida	1
BEAC	1
Febrile neutropenia	
Duration of neutropenia (day, median, range)	7 (4–35)
Duration of fever (day, median, range)	3 (1–39)
Positive blood culture finding (*n*, %)	18 (21%)
Gram-negative bacteremia (*n*, % out of positive blood culture findings)	3 (17%)
Gram-positive bacteremia (*n*, % out of positive blood culture findings)	14 (78%)
Fungal finding (*n*, % out of positive blood culture findings)	1 (5%)
Complicated course of febrile neutropenia^1^ (*n*, %)	20 (23%)
Severe sepsis^2^ (*n*, %)	8 (9%)
Septic shock^3^ (*n*, %)	3 (3%)
Fatal outcome (*n*, %)	3 (3%)

AML: acute myeloid leukemia; ASCT: autologous stem cell transplant; MM: multiple myeloma; NHL: non-Hodgkin lymphoma; HL: Hodgkin lymphoma; BEAC: carmustine, etoposide, cytarabine, and cyclophosphamide; BEAM: carmustine, etoposide, cytarabine, and melphalan; HD-MEL: high-dose melphalan; Mito-HDAraC: mitoxantrone and high-dose cytarabine; HDAraC-Ida: high-dose cytarabine and idarubicin; IAT: idarubicin, cytarabine, and thioguanine; MEA: mitoxantrone, etoposide, and cytarabine; IdAraC-Ida: intermediate-dose cytarabine and idarubicin. ^1^Febrile neutropenia with a positive blood culture finding and/or development of severe sepsis or septic shock during the period from the onset of febrile neutropenia until the recovery of neutropenia. ^2^Severe sepsis was defined as subset of sepsis with sepsis-induced organ dysfunction. ^3^Septic shock was defined as subset of sepsis with hypoperfusion (systolic arterial pressure < 90 mmHg, a mean arterial pressure < 60 mmHg, or a reduction in systolic blood pressure of >40 mmHg from baseline) despite an adequate volume resuscitation in the absence of other causes of hypotension.

**Table 2:** The levels of soluble cluster of differentiation 14 (sCD14), procalcitonin (PCT), and C-reactive protein (CRP) from day 0 to day 2 after the onset of febrile neutropenia in patients with or without septic shock. The data are expressed as medians (minimum–maximum).

	Septic shock (*n* = 3)	Without septic shock (*n* = 84)	*p* value
sCD14 (ng/L)			
Day 0	1888 (1337–3108)	1415 (349–2734)	0.176
Day 1	2550 (2249–4146)	1533 (436–2746)	0.001
Day 2	2895 (2058–3731)	1583 (655–2776)	0.044
PCT (*μ*g/L)			
Day 0	0.678 (0.138–28.6)	0.135 (0.037–1.74)	0.049
Day 1	1.07 (0.891–40.9)	0.180 (0.029–28.9)	0.007
Day 2	2.91 (1.44–4.38)	0.199 (0.036–26.9)	0.027
CRP (mg/L)			
Day 0	69 (5–212)	37 (5–286)	0.640
Day 1	226 (48–327)	73 (9–357)	0.200
Day 2	287 (231–342)	95 (7–367)	0.032

**Table 3:** The levels of soluble cluster of differentiation 14 (sCD14), procalcitonin (PCT), and C-reactive protein (CRP) from day 0 to day 2 after the onset of febrile neutropenia in patients with or without blood culture positivity. The data are expressed as medians (minimum–maximum).

	Positive blood culture finding (*n* = 18)	Negative blood culture finding (*n* = 69)	*p* value
sCD14 (ng/L)			
Day 0	1345 (349–3108)	1454 (506–2734)	0.557
Day 1	1251 (436–4146)	1578 (572–2746)	0.407
Day 2	1174 (998–3731)	1665 (655–2776)	0.059
PCT (*μ*g/L)			
Day 0	0.182 (0.058–28.6)	0.122 (0.037–1.74)	0.043
Day 1	0.519 (0.075–40.9)	0.163 (0.029–2.68)	0.001
Day 2	0.405 (0.073–26.9)	0.172 (0.036–6.13)	0.004
CRP (mg/L)			
Day 0	47 (5–286)	37 (5–245)	0.349
Day 1	98 (14–347)	68 (9–357)	0.038
Day 2	129 (28–367)	88 (7–342)	0.097

**Table 4:** The levels of soluble cluster of differentiation 14 (sCD14), procalcitonin (PCT), and C-reactive protein (CRP) from day 0 to day 2 after the onset of febrile neutropenia in patients with or without severe sepsis. The data are expressed as medians (minimum–maximum).

	Severe sepsis (*n* = 8)	No severe sepsis (*n* = 79)	*p* value
sCD14 (ng/L)			
Day 0	1315 (674–3108)	1432 (349–2734)	0.580
Day 1	1914 (869–4146)	1532 (436–2746)	0.098
Day 2	1639 (998–3731)	1605 (655–2776)	0.573
PCT (*μ*g/L)			
Day 0	0.216 (0.138–28.6)	0.122 (0.037–1.74)	0.044
Day 1	0.981 (0.154–40.9)	0.176 (0.029–28.9)	0.017
Day 2	1.44 (0.208–26.9)	0.189 (0.036–21.1)	0.028
CRP (mg/L)			
Day 0	64 (5–212)	36 (5–286)	0.237
Day 1	143 (48–327)	68 (9–357)	0.037
Day 2	231 (129–367)	86 (7–342)	0.009
